# Supplementary material for: Identification and characterization of a direct activator of a gene transfer agent
Source: Nat Commun. 2019 Feb 5;10:595. doi: 10.1038/s41467-019-08526-1 (PMC6363796; doi:10.1038/s41467-019-08526-1)
Supplement: Supplementary file 1 — Supplementary Information [file 41467_2019_8526_MOESM1_ESM.pdf]

Supplementary Information

Title: Identification and characterization of a direct activator of a gene transfer agent.

Author: Paul C.M. Fogg[1].

Supplementary Tables 1-5

Supplementary Figures 1-8

Supplementary Table 1. Upregulated genes in the RcGTA hyperproducer, DE442. List of all genes identified in the RNAseq data with a beta value (b) of 2.0 or greater, ranked from largest magnitude to lowest. Beta is the effect size of the test variable in log2 units and se_b value is the standard error. The q-value (qval) is the p-value adjusted by false discovery rate.

name	*Product*	*qval*	*b*	*se_b*
RCAP_rcc01687	phage major capsid protein; HK97 family	2.91E-65	8.61	0.49
RCAP_rcc02623	conserved hypothetical protein	5.70E-35	7.82	0.61
RCAP_rcc01688	phage conserved hypothetical protein	4.51E-32	7.68	0.63
RCAP_rcc01686	phage prohead protease; HK97 family	2.58E-41	7.54	0.54
RCAP_rcc01079	conserved hypothetical protein	1.03E-50	7.14	0.46
RCAP_rcc01080	conserved hypothetical protein	6.51E-47	7.04	0.48
RCAP_rcc01684	phage portal protein; HK97 family	2.20E-26	6.88	0.62
RCAP_rcc01691	phage major tail protein; TP901-1 family	1.32E-13	6.70	0.84
RCAP_rcc01689	phage conserved hypothetical protein	9.34E-15	6.63	0.80
RCAP_rcc02622	pyridoxamine 5'-phosphate oxidase family protein	2.90E-46	6.18	0.42
RCAP_rcc00171	conserved hypothetical protein	1.19E-40	6.02	0.44
RCAP_rcc01683	terminase-like family protein	1.20E-23	5.87	0.56
RCAP_rcc01690	conserved hypothetical protein	1.33E-08	5.72	0.92
RCAP_rcc01865	conserved hypothetical protein	2.51E-35	5.67	0.44
RCAP_rcc01695	phage conserved hypothetical protein	3.31E-07	5.64	1.00
RCAP_rcc00556	conserved hypothetical protein	6.11E-27	5.48	0.49
RCAP_rcc01682	conserved hypothetical protein	2.61E-04	5.36	1.28
RCAP_rcc01698	phage conserved hypothetical protein	7.52E-11	5.12	0.73
RCAP_rcc01866	conserved hypothetical protein	8.94E-50	5.10	0.33
RCAP_rcc01697	phage cell wall peptidase; NlpC/P60 family	2.91E-16	5.09	0.58
RCAP_rcc00555	protein of unknown function DUF847	1.31E-12	5.08	0.66
RCAP_rcc01692	conserved hypothetical protein	1.84E-08	5.08	0.82
RCAP_rcc01685	conserved hypothetical protein	1.62E-05	5.00	1.03
RCAP_rcc01699	conserved hypothetical protein	1.68E-13	4.79	0.60
RCAP_rcc01693	phage conserved hypothetical protein	1.05E-07	4.75	0.81
RCAP_rcc00203	conserved domain protein	6.56E-06	4.36	0.86
RCAP_rcc01694	phage conserved hypothetical protein	4.30E-06	4.34	0.84
RCAP_rcc01696	phage conserved hypothetical protein	8.78E-06	4.27	0.86
RCAP_rcc01701	phospholipid/glycerol acyltransferase	1.01E-11	4.07	0.55
RCAP_rcc02010	30S ribosomal protein S18, RpsR	1.37E-12	3.97	0.52
RCAP_rcc01700	serine O-acetyltransferase-1, CysE1	6.96E-09	3.84	0.60
RCAP_rcc02478	chaperonin GroL	8.43E-13	3.80	0.49
RCAP_rcc00202	ribonuclease HII, RnhB	4.34E-20	3.75	0.39
RCAP_rcc02804	conserved hypothetical protein	3.42E-14	3.73	0.46
RCAP_rcc03512	flagellar basal body-associated protein, FliL2	3.43E-18	3.65	0.40
RCAP_rcc03513	flagellar L-ring protein, FlgH	3.28E-15	3.58	0.42
RCAP_rcc00304	50S ribosomal protein L22, RplV	2.66E-09	3.55	0.55
RCAP_rcc03518	flagellar hook-basal body complex protein, FliE	8.04E-06	3.55	0.71
RCAP_rcc03519	flagellar basal-body rod protein, FlgC	5.11E-06	3.54	0.69
RCAP_rcc03516	flagellar basal-body rod protein, FlgF	1.03E-08	3.48	0.55
RCAP_rcc00367	30S ribosomal protein S16, RpsP	4.86E-19	3.42	0.36
RCAP_rcc01849	protein of unknown function DUF1457	5.42E-09	3.29	0.51
RCAP_rcc00204	conserved hypothetical protein	2.14E-15	3.29	0.39
RCAP_rcc03515	flagellar basal-body rod protein, FlgG	8.95E-14	3.28	0.41
RCAP_rcc03517	flagellar biosynthetic protein, FliQ	3.47E-05	3.25	0.69
RCAP_rcc00317	30S ribosomal protein S5, RpsE	5.44E-09	3.23	0.51
RCAP_rcc00299	50S ribosomal protein L3, RplC	3.03E-14	3.20	0.39
RCAP_rcc00290	50S ribosomal protein L9, RplI	2.35E-10	3.16	0.46
RCAP_rcc00318	50S ribosomal protein L30, RpmD	9.43E-08	3.09	0.53
RCAP_rcc00303	30S ribosomal protein S19, RpsS	3.59E-08	3.07	0.51
RCAP_rcc00490	5,10-methylenetetrahydrofolate reductase, MetF	2.99E-11	3.07	0.43
RCAP_rcc00743	ATP synthase F0 B' subunit, AtpX	1.79E-11	3.07	0.42
RCAP_rcc02436	phosphopantetheine-binding domain protein	2.74E-17	3.04	0.34
RCAP_rcc03520	flagellar basal-body rod protein, FlgB	1.54E-05	3.04	0.63
RCAP_rcc00315	50S ribosomal protein L6, RplF	5.13E-16	3.03	0.35
RCAP_rcc00537	response regulator receiver protein	3.97E-05	2.99	0.64
RCAP_rcc00486	conserved domain protein	2.12E-13	2.98	0.38
RCAP_rcc00205	conserved hypothetical protein	1.67E-13	2.98	0.38
RCAP_rcc00284	polyribonucleotide nucleotidyltransferase, Pnp	9.19E-10	2.98	0.45
RCAP_rcc03514	flagella basal body P-ring formation protein, FlgA	9.17E-15	2.97	0.36

RCAP_rcc03483	flagellar protein export ATPase, FliI	6.46E-15	2.96	0.35
RCAP_rcc00314	30S ribosomal protein S8, RpsH	2.64E-20	2.96	0.30
RCAP_rcc02361	LexA repressor	4.03E-29	2.91	0.25
RCAP_rcc00300	50S ribosomal protein L4, RplD	1.26E-13	2.89	0.36
RCAP_rcc02011	30S ribosomal protein S6, RpsF	3.67E-12	2.88	0.38
RCAP_rcc02530	light-harvesting protein B-800/850 beta chain, PucB	1.08E-07	2.87	0.49
RCAP_rcc00042	PAS/PAC sensor domain protein	7.59E-04	2.86	0.73
RCAP_rcc00289	50S ribosomal protein L10, RplJ	1.24E-08	2.86	0.46
RCAP_rcc01896	transcriptional regulator; GntR family	2.37E-12	2.85	0.38
RCAP_rcc00305	30S ribosomal protein S3, RpsC	9.68E-07	2.85	0.52
RCAP_rcc01762	chemotaxis protein, CheY2	1.32E-06	2.83	0.53
RCAP_rcc01830	fructose-bisphosphate aldolase, Fba	1.56E-11	2.82	0.39
RCAP_rcc00290	50S ribosomal protein L7/L12, RplL	6.48E-07	2.79	0.51
RCAP_rcc00313	30S ribosomal protein S14, RpsN	7.42E-24	2.77	0.26
RCAP_rcc00744	ATP synthase F0 B subunit, AtpF	9.33E-13	2.76	0.36
RCAP_rcc00306	50S ribosomal protein L16, RplP	8.48E-09	2.73	0.43
RCAP_rcc02251	membrane protein; putative	2.26E-07	2.73	0.48
RCAP_rcc00482	conserved hypothetical protein	4.67E-10	2.72	0.40
RCAP_rcc01761	conserved hypothetical protein	1.79E-06	2.70	0.51
RCAP_rcc02531	light-harvesting protein B-800/850 alpha chain, PucA	3.85E-06	2.69	0.52
RCAP_rcc01963	conserved hypothetical protein	6.99E-08	2.69	0.45
RCAP_rcc00316	50S ribosomal protein L18, RplR	4.16E-11	2.68	0.38
RCAP_rcc00287	50S ribosomal protein L11, RplK	1.01E-12	2.68	0.35
RCAP_rcc03480	flagellar motor switch protein, FliN	3.52E-03	2.65	0.76
RCAP_rcc00288	50S ribosomal protein L1, RplA	3.48E-12	2.63	0.35
RCAP_rcc00172	glycosyl transferase family protein	1.44E-08	2.60	0.42
RCAP_rcc01702	phosphatidate cytidylyltransferase-2, CdsA2	4.20E-05	2.60	0.56
RCAP_rcc00283	conserved hypothetical protein	3.07E-25	2.60	0.24
RCAP_rcc01363	2-isopropylmalate synthase, LeuA	1.25E-10	2.60	0.37
RCAP_rcc02067	methyltransferase; type 11 family	8.96E-11	2.58	0.37
RCAP_rcc03209	protein containing DUF484	3.68E-06	2.56	0.50
RCAP_rcc02165	ribonuclease E, Rne	2.04E-09	2.56	0.39
RCAP_rct00033	unknown	3.42E-07	2.55	0.45
RCAP_rcc01766	chemotaxis protein, CheY3	1.04E-05	2.54	0.51
RCAP_rcc03050	50S ribosomal protein L25, RplY	3.00E-09	2.50	0.39
RCAP_rcc02069	TPR repeat domain protein	2.26E-07	2.49	0.43
RCAP_rcc00007	flagellar hook protein, FlgE	1.22E-03	2.48	0.66
RCAP_rcc01623	transcriptional regulator; AraC family	8.43E-13	2.48	0.32
RCAP_rcc00499	conserved hypothetical protein	1.41E-04	2.46	0.56
RCAP_rcc03208	tyrosine recombinase, XerC	1.11E-04	2.45	0.56
RCAP_rcc01057	gas vesicle protein, GvpG	5.29E-11	2.44	0.34
RCAP_rcc03486	chemotaxis protein, MotA	1.21E-06	2.44	0.45
RCAP_rcc03482	flagellar M-ring protein, FliF	1.51E-10	2.42	0.35
RCAP_rcc00301	50S ribosomal protein L23, RplW	2.98E-09	2.41	0.37
RCAP_rcc00312	50S ribosomal protein L5, RplE	7.23E-29	2.40	0.21
RCAP_rcc03452	sensor histidine kinase/response regulator receiver protein	3.39E-12	2.39	0.32
RCAP_rcc01214	ketol-acid reductoisomerase, IlvC	2.08E-08	2.37	0.39
RCAP_rcc02437	cytochrome P450 family protein	9.13E-18	2.33	0.26
RCAP_rcc02980	conserved hypothetical protein	1.11E-11	2.32	0.32
RCAP_rcc00296	translation elongation factor G-1, FusA	1.66E-05	2.32	0.48
RCAP_rcc02591	surface presentation of antigens protein family	1.80E-05	2.31	0.48
RCAP_rcc01369	ABC transporter; periplasmic substrate-binding protein	2.62E-08	2.29	0.38
RCAP_rcc01867	protein of unknown function DUF853; NPT hydrolase putative	4.76E-08	2.29	0.38
RCAP_rcc01674	glutamine synthetase-3, GlnA3	2.11E-07	2.29	0.40
RCAP_rcc01940	hemolysin-type calcium-binding repeat family protein	9.32E-21	2.28	0.23
RCAP_rcc03479	flagellar biosynthetic protein, FliP	2.88E-04	2.28	0.55
RCAP_rcc00326	DNA-directed RNA polymerase alpha subunit, RpoA	9.39E-08	2.27	0.39
RCAP_rcc03485	conserved hypothetical protein	3.71E-05	2.23	0.48
RCAP_rcc00056	flagellar biosynthesis protein, FlhA	2.73E-08	2.23	0.37
RCAP_rcc00335	glutamate/aspartate ABC transporter; periplasmic glutamate/aspartate-binding protein, BztA	2.60E-05	2.21	0.47
RCAP_rcc03526	conserved hypothetical protein	4.39E-03	2.21	0.65
RCAP_rcc03483	flagellar basal body-associated protein, FliL1	8.35E-05	2.19	0.49
RCAP_rcc03113	conserved hypothetical protein	2.00E-03	2.19	0.60
RCAP_rcc00327	50S ribosomal protein L17, RplQ	7.90E-07	2.18	0.40
RCAP_rcc01405	hypothetical protein	3.83E-07	2.17	0.39
RCAP_rcc00742	ATP synthase F0 C subunit, AtpE	4.23E-05	2.16	0.47
RCAP_rcc03524	flagellar FlaF family protein	5.13E-05	2.16	0.47
RCAP_rcc02597	hypothetical protein	3.43E-06	2.16	0.42
RCAP_rcc01138	conserved hypothetical protein	4.97E-13	2.15	0.28

RCAP_rcc01184	CsbD family protein	5.84E-09	2.14	0.34
RCAP_rcc00297	translation elongation factor Tu-2, Tuf2	1.42E-04	2.14	0.49
RCAP_rcc02971	ATP synthase F1 beta subunit, AtpD	3.10E-05	2.14	0.46
RCAP_rcc03484	conserved hypothetical protein	4.27E-03	2.13	0.63
RCAP_rcc01765	chemotaxis protein, CheA2	3.72E-04	2.12	0.52
RCAP_rcc00292	DNA-directed RNA polymerase beta' subunit, RpoC	1.93E-04	2.12	0.50
RCAP_rcc01763	chemotaxis protein methyltransferase, CheR3	4.87E-06	2.12	0.41
RCAP_rcc00048	metal dependent phosphohydrolase	5.40E-05	2.10	0.46
RCAP_rcc01703	pyruvate dehydrogenase complex; E2 component; dihydrolipoyllysine-residue acetyltransferase, PdhC	1.29E-06	2.10	0.39
RCAP_rcc03529	flagellar hook capping protein, FlgD	3.87E-04	2.08	0.51
RCAP_rcc00295	30S ribosomal protein S7, RpsG	4.32E-05	2.08	0.45
RCAP_rcc02609	ATP-dependent Clp protease; proteolytic subunit, ClpP	2.38E-04	2.08	0.49
RCAP_rcc03525	flagellin protein, FlaA	4.06E-03	2.06	0.60
RCAP_rcc03527	flagellar protein FlgJ; putative	7.15E-05	2.05	0.45
RCAP_rcc02972	ATP synthase F1; gamma subunit, AtpG	1.86E-07	2.04	0.35
RCAP_rcc01726	methyl-accepting chemotaxis protein, McpH	1.29E-07	2.04	0.35
RCAP_rcc00557	methionyl-tRNA synthetase, MetG	5.42E-09	2.02	0.32
RCAP_rcc03523	flagellin synthesis repressor protein FlbT; putative	8.86E-04	2.02	0.52
RCAP_rcc01221	conserved hypothetical protein	5.79E-09	2.01	0.32
RCAP_rcc02768	ubiquinol--cytochrome-c reductase; iron-sulfur subunit, PetA	6.48E-05	2.01	0.44
RCAP_rcc01822	translation elongation factor Ts, Tsf	4.43E-06	2.00	0.39
RCAP_rcc00720	succinyl-CoA synthetase (ADP-forming); beta subunit, SucC	1.13E-03	2.00	0.53
RCAP_rcc01240	cytochrome c2-1, CycA1	8.63E-06	2.00	0.40

Supplementary Table 2. Downregulated genes in the RcGTA hyperproducer, DE442. List of all genes identified in the RNAseq data with a beta value (b) of -2.0 or greater, ranked from lowest magnitude to largest. Beta is the effect size of the test variable in log2 units and se_b value is the standard error. The q-value (qval) is the p-value adjusted by false discovery rate.

name	*product*	*qval*	*b*	*se_b*
RCAP_rcc01871	acyl carrier protein-2, AcpP2	2.03E-06	-2.05	0.39
RCAP_rcc01016	Unknown		-2.06	
RCAP_rcc02566	reverse transcriptase catalytic domain protein	1.45E-05	-2.07	0.42
RCAP_rcc02637	RNA polymerase sigma factor%2C sigma-70 family%2C ECF subfamily	1.85E-01	-2.07	1.18
RCAP_rcc02550	translation initiation factor IF-1, InfA	1.20E-03	-2.11	0.56
RCAP_rcc03248	metallophosphoesterase family protein	8.81E-08	-2.12	0.36
RCAP_rcc00559	phosphatidylethanolamine N-methyltransferase, PmtA	2.40E-07	-2.13	0.37
RCAP_rcc02871	glycolate dehydrogenase subunit, GlcD	8.33E-07	-2.16	0.40
RCAP_rcp00036	unknown	2.94E-10	-2.17	0.32
RCAP_rcc00028	isopentenyl-diphosphate delta-isomerase-1, Idi1	4.51E-06	-2.25	0.44
RCAP_rcc00274	protein of unknown function DUF1674	1.67E-07	-2.25	0.39
RCAP_rcc03431	branched-chain amino acid ABC transporter, periplasmic branched-chain amino acid binding protein, LivK2	1.02E-05	-2.26	0.46
RCAP_rcc01020	diguanylate cyclase/phosphodiesterase family protein	7.49E-15	-2.27	0.27
RCAP_rcc01888	conserved hypothetical protein	3.27E-03	-2.31	0.66
RCAP_rcc03112	conserved hypothetical protein	1.74E-04	-2.31	0.54
RCAP_rcc01038	conserved domain protein	2.31E-06	-2.31	0.44
RCAP_rcc01167	ATP-dependent Clp protease adaptor protein, ClpS	3.20E-07	-2.33	0.41
RCAP_rcc03428	branched-chain amino acid ABC transporter/permease protein, LivH3	7.98E-10	-2.34	0.35
RCAP_rcc02151	methyl-accepting chemotaxis sensory transducer	3.56E-17	-2.36	0.26
RCAP_rcc00357	macrolide export ABC transporter, ATP-binding/permease protein, MacB	8.87E-05	-2.41	0.54
RCAP_rct00010	unknown	4.55E-09	-2.43	0.38
RCAP_rcc02811	RNA polymerase sigma-32 factor-2, RpoH2	5.23E-05	-2.55	0.56
RCAP_rcc01035	conserved domain protein	1.77E-08	-2.56	0.41
RCAP_rcc02681	cytochrome b561 family protein	9.85E-03	-2.97	0.95
RCAP_rcc00524	glyoxylate reductase-1, GyaR1	2.11E-07	-3.02	0.53
RCAP_rcc02682	cytochrome c'	1.98E-08	-3.04	0.49
RCAP_rcc01037	conserved domain protein	8.97E-18	-3.05	0.33
RCAP_rcc02323	hypothetical protein	1.84E-08	-3.07	0.50
RCAP_rcc01447	5-aminolevulinate synthase, HemA	6.53E-12	-3.17	0.43
RCAP_rcc02638	calcium-binding EF-hand domain protein	3.06E-03	-3.22	0.92
RCAP_rcc02367	calcium-binding protein	2.87E-07	-3.43	0.60
RCAP_rcc00358	macrolide export ABC transporter, macrolide-specific efflux protein, MacA	9.80E-13	-3.89	0.51
RCAP_rcc00562	molybdenum ABC transporter, periplasmic molybdenum-binding protein, ModA1	1.63E-65	-3.98	0.23
RCAP_rcc00353	membrane protein%2C putative	8.04E-06	-4.49	0.90
RCAP_rcc01269	conserved domain protein	4.90E-24	-4.52	0.43
RCAP_rcp00062 (Plasmid)	unknown	8.69E-94	-10.99	0.52
RCAP_rcp00122 (Plasmid)	malonate transporter subunit, MadM	1.66E-86	-11.39	0.57

Supplementary Table 3. Abundance of RcGTA-related transcripts in the RcGTA hyper producer. 29 upregulated genes in DE442 have a b-value of 4 or above, and contain all of the genes from the core RcGTA gene cluster (dark blue), known RcGTA genes from elsewhere in the genome (light blue). Hypothetical proteins are unshaded and *gafA* is highlighted in orange. In grey are genes known to be involved in RcGTA production/receipt or the CtrA regulon. Abundance was ranked based on the beta value and the rank of each gene in the entire dataset is provided. Beta is the effect size of the test variable in log2 units and se_b value is the standard error. The q-value (qval) is the p-value adjusted by false discovery rate.

RANK	NAME	PRODUCT	QVAL	B	SE_B
**1**	*RCAP_rcc01687*	GTA major capsid protein	2.91E-65	8.61	0.49
**2**	*RCAP_rcc02623*	Conserved hypothetical protein	5.70E-35	7.82	0.61
**3**	*RCAP_rcc01688*	GTA hypothetical protein	4.51E-32	7.68	0.63
**4**	*RCAP_rcc01686*	GTA prohead protease	2.58E-41	7.54	0.54
**5**	*RCAP_rcc01079*	GTA headspike protein	1.03E-50	7.14	0.46
**6**	*RCAP_rcc01080*	GTA headspike protein	6.51E-47	7.04	0.48
**7**	*RCAP_rcc01684*	GTA portal protein	2.20E-26	6.88	0.62
**8**	*RCAP_rcc01691*	GTA major tail protein	1.32E-13	6.70	0.84
**9**	*RCAP_rcc01689*	GTA hypothetical protein	9.34E-15	6.63	0.80
**10**	*RCAP_rcc02622*	Conserved hypothetical protein	2.90E-46	6.18	0.42
**11**	*RCAP_rcc00171*	GTA tailspike protein	1.19E-40	6.02	0.44
**12**	*RCAP_rcc01683*	GTA large terminase subunit	1.20E-23	5.87	0.56
**13**	*RCAP_rcc01690*	GTA hypothetical protein	1.33E-08	5.72	0.92
**14**	*RCAP_rcc01865*	GTA activation factor A (GafA)	2.51E-35	5.67	0.44
**15**	*RCAP_rcc01695*	GTA hypothetical protein	3.31E-07	5.64	1.00
**16**	*RCAP_rcc00556*	Holin	6.11E-27	5.48	0.49
**17**	*RCAP_rcc01682*	GTA hypothetical protein	2.61E-04	5.36	1.28
**18**	*RCAP_rcc01698*	GTA hypothetical protein	7.52E-11	5.12	0.73
**19**	*RCAP_rcc01866*	GTA maturation protein	8.94E-50	5.10	0.33
**20**	*RCAP_rcc01697*	GTA cell wall peptidase	2.91E-16	5.09	0.58
**21**	*RCAP_rcc00555*	Endolysin	1.31E-12	5.08	0.66
**22**	*RCAP_rcc01692*	GTA hypothetical protein	1.84E-08	5.08	0.82
**23**	*RCAP_rcc01685*	GTA hypothetical protein	1.62E-05	5.00	1.03
**24**	*RCAP_rcc01699*	Conserved hypothetical protein	1.68E-13	4.79	0.60
**25**	*RCAP_rcc01693*	GTA hypothetical protein	1.05E-07	4.75	0.81
**26**	*RCAP_rcc00203*	Conserved domain protein	6.56E-06	4.36	0.86
**27**	*RCAP_rcc01694*	GTA hypothetical protein	4.30E-06	4.34	0.84
**28**	*RCAP_rcc01696*	GTA hypothetical protein	8.78E-06	4.27	0.86
**29**	*RCAP_rcc01701*	Phospholipid/glycerol acyltransferase	1.01E-11	4.07	0.55
**63**	*RCAP_rcc02361*	LexA repressor	4.03E-29	2.91	0.25
**67**	*RCAP_rcc00042*	DivL, PAS/PAC sensor	7.59E-04	2.86	0.73
**141**	*RCAP_rcc02609*	ClpP protease, proteolytic subunit	2.38E-04	2.08	0.49
**289**	*RCAP_rcc01663*	CtrA, cell cycle transcriptional regulator	0.13	1.32	0.67
**440**	*RCAP_rcc02608*	ClpX protease, ATP-binding subunit	0.12	0.91	0.46
**825**	*RCAP_rcc03144*	GcrA, cell cycle regulator	0.19	0.45	0.26
**956**	*RCAP_rcc00460*	ComM, competence protein	0.30	0.35	0.25
**1098**	*RCAP_rcc00328*	GtaR, LuxR-family transcriptional regulator	0.38	0.26	0.21
**1511**	*RCAP_rcc03098*	DprA, DNA protecting protein	0.95	0.05	0.56
**1696**	*RCAP_rcc00222*	RadC, DNA repair protein	0.94	-0.04	0.37
**1735**	*RCAP_rcc00645*	Diguanylate cyclase, PAS/PAC sensor	0.93	-0.06	0.42
**1984**	*RCAP_rcc01749*	CckA, Signal transduction histidine kinase	0.64	-0.17	0.25
**2575**	*RCAP_rcc01751*	RecA, SOS response protein	0.55	-0.43	0.50
**2917**	*RCAP_rcc00329*	GtaI, HSL autoinducer synthesis protein	0.01	-0.58	0.19
**2926**	*RCAP_rcc02362*	Competence protein	0.15	-0.59	0.31

Supplementary Table 4. List of primers used in this study.

Name	Sequence (5' to 3')	Application
**Cloning Primers**		
pPuf F	CGACTCTAGAGGATCCGAGCTTCGGAATCTGCG	Overexpression promoter
pPuf R	CATAACAACCTCCGGATTGGCAGAC	
CtrA(H6) F	TTTCAGGGCGCCATGCGGATTTTGTTGGTGG	Construction of pCMF181
CtrA(H6) R	CCGATATCAGCCATGGGAGCGTCCAGAGAGAAGC	
CtrA(pPuf) F	CCGGAGGTTGTTATGCGGATTTTGTTGGTGG	Construction of pCMF182
CtrA(66T) R	CGGTACCCGGGGATCGGAGCGTCCAGAGAGAAGC	
CtrA D51E F	TCCTTCTCGAACTGAACCTTCCCGACATG	Site directed mutagenesis to make pCMF184 & 5
CtrA D51E R	AGGTTCAGTTCGAGAAGGATCAGGTCGTAA	
CtrA D51A F	TCCTTCTCGCTCTGAACCTTCCCGACATG	Site directed mutagenesis to make pCMF192 & 4
CtrA D51A R	AGGTTCAGAGCGAGAAGGATCAGGTCGTAA	
H6-gafA F	TTTCAGGGCGCCATGAAGACCATGCAAGATC	Construction of pCMF176
H6-gafA R	CCGATATCAGCCATGGATGCAAATTGCCTGTGC	
pP-gafA F	CCGGAGGTTGTTATGAAGACCATGCAAGATC	Construction of pCMF177
pP-gafA R	CGGTACCCGGGGATCGATGCAAATTGCCTGTGC	
MBP-gafA F	TCCAGGGACCAGCAATGAAGACCATGCAAGATC	Construction of pCMF193
MBP-gafA R	TGAGGAGAAGGCGCGGATGCAAATTGCCTGTGC	
171_gafA F	AGATCTGGATCCATAATGGGTAACTGGGCAGAAAAC	Construction of pCMF196
171_gafA R	TATCAACCGCCCATAAATCACCCTTGGCGACAACC	
170_gafA F	AGATCTGGATCCATAATGATGCAGGGGACGGGAC	Construction of pCMF195
170_gafA R	TATCAACCGCCCATAGTGCCGTGGTGGCCTATGG	
GtaR(H6) F	TTTCAGGGCGCCATGTCCATACACACCGAA	Construction of pCMF199
GtaR(H6) R	CCGATATCAGCCATGGAAGTGACCGCTGCGAGG	
CleF	CGCGCCTTCTCCTCACATATGGCTAGC	Construction of pCMF193
CleR	TTGCTGGTCCCTGGAACAGAACTTCC	
**Gene Knock-Out Primers**		
CtrA ext F	CGACTCTAGAGGATCACAGTCCTTTAACCAGACC	Deletion and replacement of ctrA with gentamicin^R
CtrA ext R	CGGTACCCGGGGATCGTAGCGCAAGGACAGCGAG	
CtrA inv F	AACAATTCGTTCAAGGATCAGGGCGACCTTGACC	
CtrA inv R	GGGAATCAGGGGATCCCACCAACAAAATCCGCAT	
gafA ext F	CGACTCTAGAGGATCAGGAAGCCCTTGCCATAGG	Deletion and replacement of gafA with gentamicin^R
gafA ext R	CGGTACCCGGGGATCGCGAAGCTGGAGTTCAACC	
gafA inv R	GGGAATCAGGGGATCCGCCATTATGACGAAACC	
gafA inv F	AACAATTCGTTCAAGGGTCGAGGCGTGACAGC	
Gent R	CTTGAACGAATTGTTAGG	Gentamicin resistance
Gent F	GATCCCCTGATTCCCTTTGT	
**Site Directed Mutagenesis Primers**		
pGafA_M1 F	CCCCTTGGTTGTGAAATGGGGCGATCTCGAAAATTCT	Mutation of CtrA binding site (TTAAC to ACAAC)
pGafA_M1 R	GAGATCGCCCCATTTCACAACCAAGGGGTAACCAAAG	
pGafA_M2 F	TTGCTTTGGTTGTCCCTTGGTTAAGAAATGGGGCGAT	Mutation of CtrA binding site (GTAAC to ACAAC)
pGafA_M2 R	TCTTAACCAAGGGACAACCAAAGCAACCCACGGT	

EMSA Probe Primers

pGTA5	GGGATGCGGCTGCAGACCGATCC	Creation of PCR products for band shifts
pGTA R	GCTGACCATCGCCAGGGCCAGTTCC	
pGafA F	CCGGATCAGCAGTTTCGGAC	
pGafA inv R	GGGAATCAGGGGATCCGCCATTATGACGAAACC	
555 F (Kan)	TAATCGCGGCCTCGAATCGTCATCGACCTGAAGGC	
555 R (Kan)	ATTTTGAGACACAACCGAAATCAGGTTAACGATCC	
pGafA_50	TCGAGATCGCCCCATTTCTTAACCAAGGGGTAACCAAAGCAACCCACGGT	50 bp complimentary oligonucleotides
pGafA_50 RC	ACCGTGGGTTGCTTTGGTTACCCCTTGGTTAAGAAATGGGGCGATCTCGA	
pCtrA_50	GAACGGAATCCTGCGACAGTAACTACGTGTTAACCATTTGGTGACAAGGT	
pCtrA_50 RC	ACCTTGTCACCAAATGGTTAACACGTAGTTACTGTCGCAGGATTCCGTTC	
pGTA1	TCCGGGGTTTTTTCTTTTCAGCGGGTGCAACCCTGAATATAGCACTTGAC	
pGTA1 RC	GTCAAGTGCTATATTCAGGGTTGCACCCGCTGAAAAGAAAAAACCCCGGA	
pGTA2	TTTGCGAACGCTTCAAGGTAGAGATAAGGCATGCTAGGAGAGGTGGGCAA	
pGTA2 RC	TTGCCCACCTCTCCTAGCATGCCTTATCTCTACCTTGAAGCGTTCGCAAA	
pGTA3	GCGCCGCGGGTGACCGTGTGCGCTTTTTTCATTTCGCTCGTGCGGACAGG	
pGTA3 RC	CCTGTCCGCACGAGCGAAATGAAAAAAGCGCACACGGTCACCCGCGGCGC	
pGTA4	TTCATTTCGCTCGTGCGGACAGGCATGAGAGGCGGGTCACGCAAGACATG	
pGTA4 RC	CATGTCTTGCGTGACCCGCCTCTCATGCCTGTCCGCACGAGCGAAATGAA	
pGTA5	GGCATGCTAGGAGAGGTGGGCAAGCGCCGCGGGTGACCGTGTGCGCTTTT	
pGTA5 RC	AAAAGCGCACACGGTCACCCGCGGCGCTTGCCCACCTCTCCTAGCATGCC	
pGafA2	TGCAGATGCCGTTTGCGCTATCTGACTGCCAGCCTGTCGATGGGAGACCGGA GATGAAGA	
pGafA RC	TCTTCATCTCCGGTCTCCCATCGACAGGCTGGCAGTCAGATAGCGCAAACGG CATCTGCA	
NS_50	CGGGCTGAGATCGGGCGCCGCCTGGCTTGCCTGCGCGACGCCGCAGGAGG	
NS_50 RC	CCTCCTGCGGCGTCGCGCAGGCAAGCCAGGCGGCGCCCGATCTCAGCCCG	

qPCR Primers

gafA qF	GCTGAACGGCTGGATCTT	Quantification of *gafA* transcripts
gafA qR	TTCCAACAGCCGCTTCAA	
ctrA qF	TTTGGCGCCGATGATTAC	Quantification of *ctrA* transcripts
ctrA qR	GGATGATCGACTGCGAATG	
terL qF	CGACGAGCTGGCGAAAT	Quantification of terminase transcripts
terL qR	TTGTTGAGGATCGCCTTCAG	
g5 qF	GGTCGTGGATCTGGTCTAT	Quantification of capsid transcripts
g5 qR	GTCGGCATCCTTCATCTTG	
555 qF	CTGGGCCTTGATCTGAACC	Quantification of endolysin transcripts
555 qR	GTTTCGGCGCCTTGAAATAC	
divL qF	CGACGCTTTATGCCTTTCT	Quantification of *divL* transcripts
divL qR	GTTCCAGTTCCGTCATCTTC	
lexA qF	GCATTACGCGCTTGAAGT	Quantification of *lexA* transcripts
lexA qR	ACGACGATATCGCCATTCT	
uvrD qF	CAGAAGGAACACACGGTCAA	Quantification of *uvrD* reference gene transcripts
uvrD qR	AAAGTGTCAGGCGGAATCTC	
pGafA qF	TGGGAGACCGGAGATGA	Quantification of native *gafA* transcripts
pGafA qR	CGCCAGCTGCCGGATCG	
pCtrA qF	GCGGAGAACCCAGGATG	Quantification of native *ctrA* transcripts
pCtrA qR	ACGTTGAGGTTGGCATG	

Supplementary Table 5. List of plasmids used in this study.

Plasmid	Description	Reference
pCM66T	pCM66T: trimmed pCM66 backbone was a gift from Mary Lidstrom<br>Broad host range vector; ColE1, OriV, IncP/traJ, Kanamycin^R	Addgene plasmid # 74738
pSRKBB	pSRKBB-empty was a gift from Claudia Schmidt-Dannert<br>Broad host range vector; rep/per, pBR322, Kanamycin^R	Addgene plasmid # 59449
pETFPP_2	Expression vector; T7 promoter-His6-MBP-3c Cleavage Site, Kanamycin^R	[1]
pEHisTev	Expression vector; T7 promoter-His6 tag, Kanamycin^R	[2]
**CtrA Constructs**		
pCMF181	pEHisTEV::His6-*ctrA*	This Study
pCMF182	pCM66T::pPuf-*ctrA*	This Study
pCMF184	pEHisTEV::His6-*ctrA* D51E (phosphorylation mimic)	This Study
pCMF185	pCM66T::pPuf-*ctrA* D51E (phosphorylation mimic)	This Study
pCMF190	pCM66T::*ctrA*Δgentamycin^R	
pCMF192	pEHisTEV::His6-*ctrA* D51A (non-phosphorylatable mutant)	This Study
pCMF194	pCM66T::pPuf-*ctrA* D51A (non-phosphorylatable mutant)	This Study
**GafA Constructs**		
pCMF176	pEHisTEV::His6-*gafA*	This Study
pCMF177	pCM66T::pPuf-*gafA*	This Study
pCMF180	pCM66T::*gafA* plus flanking DNA	This Study
pCMF188	pCM66T::*gafA*Δgentamycin^R	This Study
pCMF193	pETFPP_2::His6-MBP-*gafA*	This Study
pCMF195	pLac'-1865 homologue (DSM15170)	This Study
pCMF196	pLac'-1865 homologue (DSM15171)	This Study
pCMF214	pCMF180 mutated TTAAC to ACAAC CtrA binding site	This Study
pCMF215	pCMF180 mutated GTAAC to ACAAC CtrA binding site	This Study
**GtaR Constructs**		
pCMF199	pEHisTEV::His6-*gtaR*	This Study

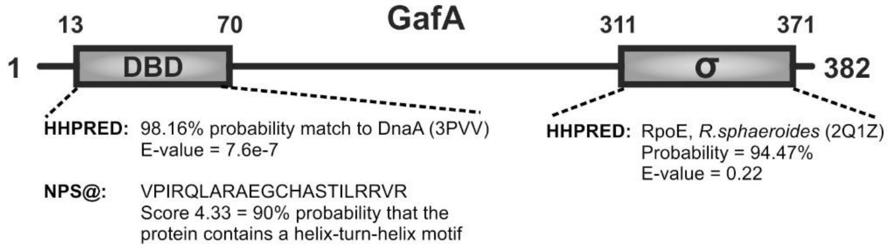

Supplementary Figure 1. Schematic of GTA activation factor protein (GafA). The predicted DNA binding domain (DBD) and sigma factor domain (σ) are indicated as grey boxes with amino acid boundaries for each feature annotated. Structural comparisons were carried out with HHPRED [3,4] using the PDB database. For each domain, the top hit is annotated below the figure along with the associated probability score and E-value. The DBD is also predicted to contain a helix-turn-helix motif (HTH) [5], the amino acid sequence of which is provided as identified by both NPS@: Network protein sequence analysis [6] and GYM 2.0 [7]. The score and probability values from NPS@ are annotated.

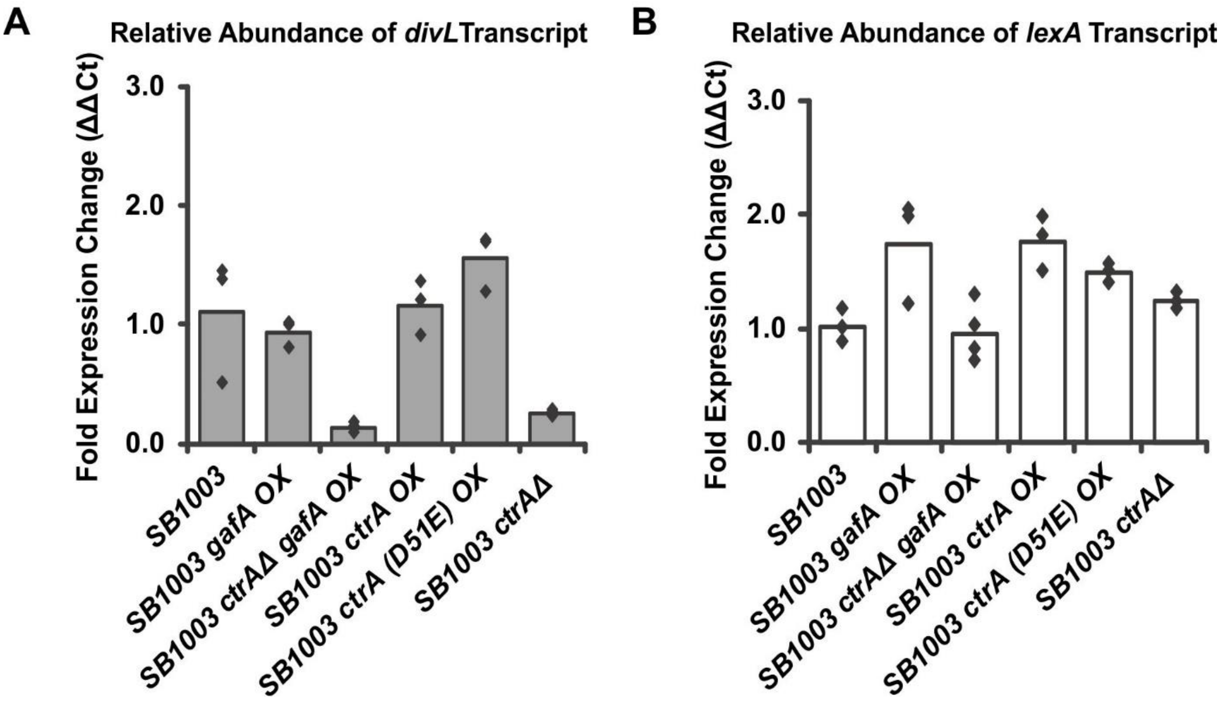

Supplementary Figure 2. Relative Transcription of *lexA* (A) and *divL* (B). The *R. capsulatus* strains assessed are annotated below each graph. All Y-axis fold expression changes are normalized using *uvrD* as an endogenous reference gene (ΔCt) and relative to the wild-type SB1003 strain (ΔΔCt). Individual replicates are shown as diamonds (n=3 for all samples, except SB1003 *ctrA*Δ *gafA* OX where n=4). Source data are provided as a Source Data file.

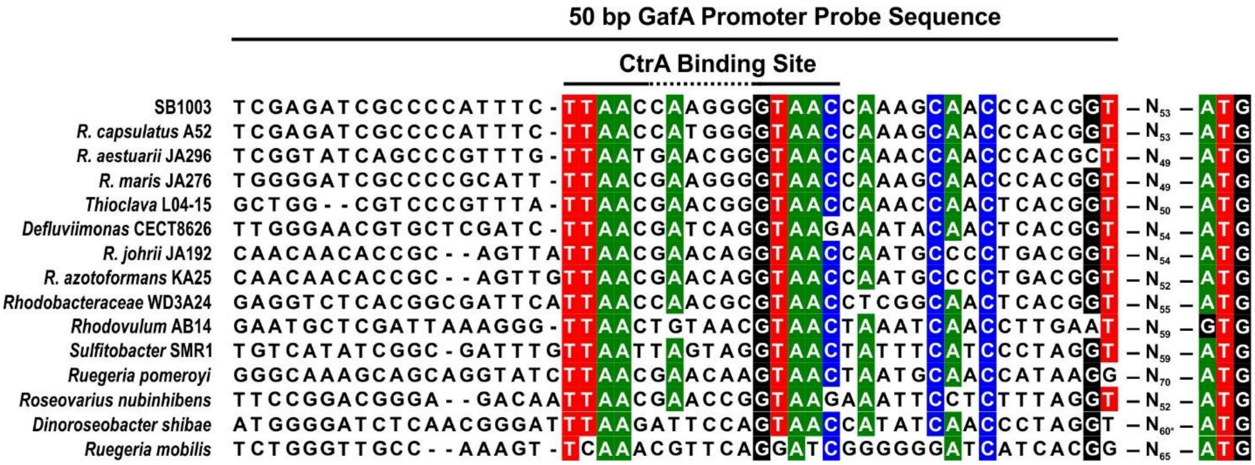

Supplementary Figure 3. Alignment of the *R. capsulatus gafA* promoter to comparable promoters from 14 species. GafA was queried against the Genbank nr protein sequences database (access date 14th June 2018) using Blastp and the results returned were condensed by selection of the top hit belonging to each of the first ten different species matches. The promoter region for each protein was identified and compared to the *gafA* promoters from four experimentally proven *Rhodobacteraceae* GTA producers. Alignment was produced using ClustalW2 [8] and an 80% cut-off for conservation shading is shown. The two half-sites of the CtrA binding site are shown as solid lines and the spacer region as a dotted line. The ATG start codon is depicted to the right with distance to the binding region indicated.

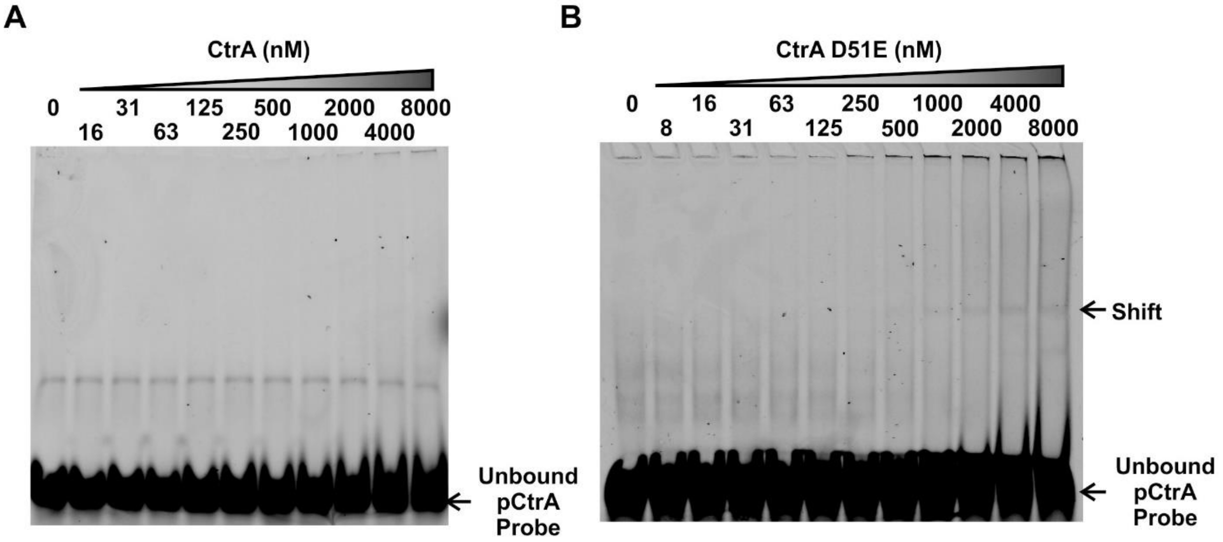

Supplementary Figure 4. CtrA/CtrA^{D51E} Promoter Binding Activity. Band shifts of Cy5-labelled *ctrA* promoter DNA (pCtrA) incubated with either WT CtrA (**A**) or phosphomimetic CtrA^{D51E} (**B**) protein at the concentrations specified above each image. Source data are provided as a Source Data file.

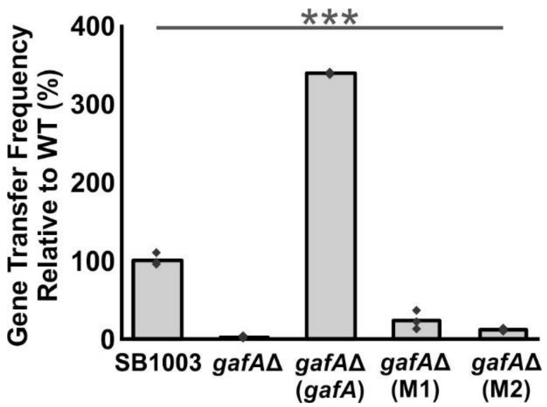

Supplementary Figure 5. Mutation of the CtrA binding site in the GafA promoter impairs GTA expression. GTA gene transfer assays for *R. capsulatus* SB1003, *gafA* knock-out (*gafA*Δ), *gafA* knock-out complemented *in trans* with *gafA* expressed from its native promoter (*gafA*Δ(*gafA*)), and *gafA* knock-out complemented *in trans* with *gafA* expressed from its native promoter with one CtrA binding site mutated from TTAAC to ACAAC (*gafA*Δ(M1)) or one CtrA binding site mutated from GTAAC to ACAAC (*gafA*Δ(M2)). Individual replicates are shown as diamonds (n= 3), One Way ANOVA significance versus the control (SB1003) is indicated above the chart (*** = p<0.001). Source data are provided as a Source Data file.

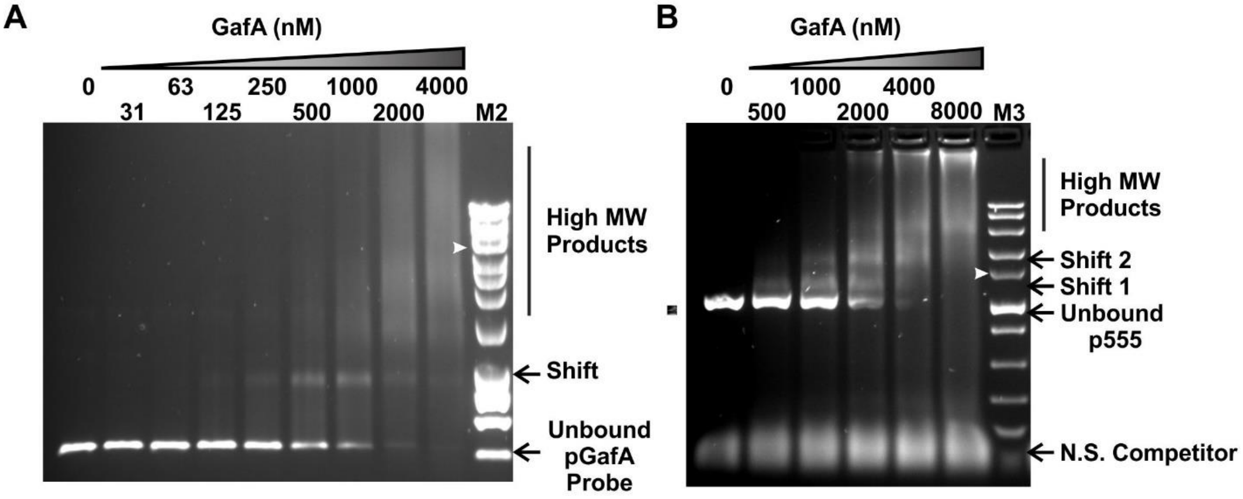

Supplementary Figure 6. Band shifts of GafA vs the *gafA* (A, pGafA) and RcGTA endolysin promoters (B, p555). Concentration of GafA is shown above each image. DNA probes were PCR amplicons of 420 bp and 1,047 bp for pGafA and p555, respectively. Unbound probe DNA and Protein:DNA complexes (Shift) are annotated. 50 bp dsDNA oligos (N.S. Competitor) were included in molar excess as a non-specific competitor. At high protein concentrations some high molecular weight complexes were observed in the wells and some smearing possibly indicative of precipitation or higher order complexes. Bioline HyperLadder 1 kb DNA ladder (M2) or NEB 1 kb DNA Ladder (M3) were used; the 4 kb band is annotated with a white arrow head. Source data are provided as a Source Data file.

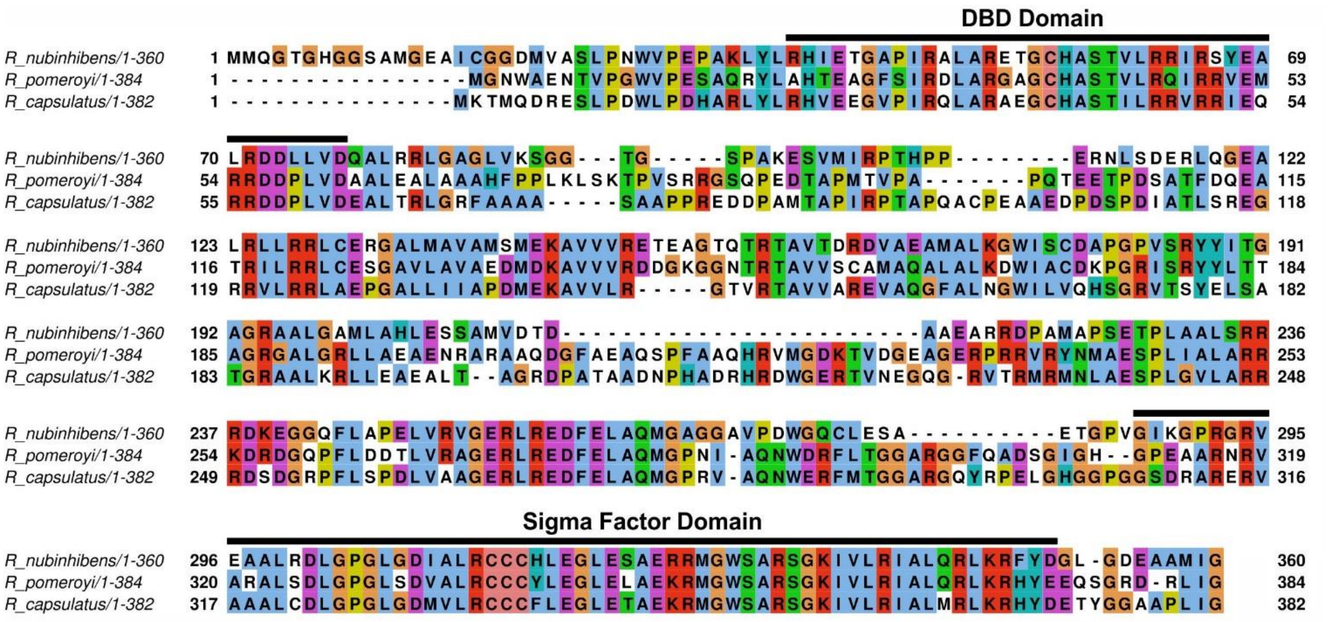

Supplementary Figure 7. Alignment of GafA from the three GTA producing species used in this study. Protein sequences were aligned with ClustalΩ [9] and similarity shading uses the ClustalX coloring scheme in Jalview [10]. The DNA binding domain (DBD) and sigma factor domain are indicated by solid black lines and are annotated.

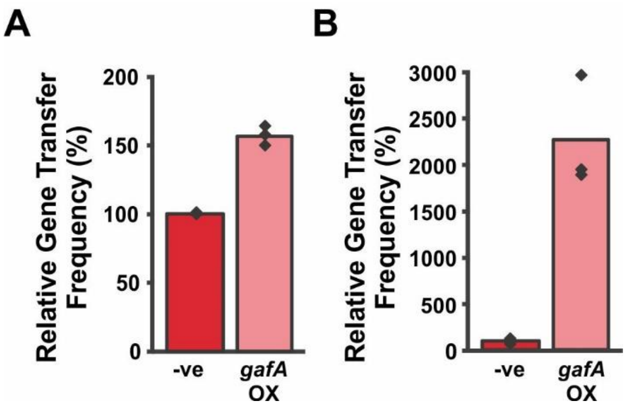

Supplementary Figure 8. The effect of *Roseovarius nubinhibens gafA* overexpression on transfer of antibiotic resistance by two non-*Rhodobacter* species. A, *In vitro* GTA assay of spontaneous streptomycin resistant *Roseovarius nubinhibens* clones containing the pCMF195 *gafA* overexpression construct (*gafA* OX) or without (-ve). The antibiotic sensitive parental strain was used as a recipient. Individual replicates are shown as diamonds (n= 3) **B,** Co-culture GTA assay of *Ruegeria mobilis* spontaneous rifampicin and streptomycin resistant clones containing the pCMF195 *gafA* overexpression construct (*gafA* OX) or without (-ve). Source data are provided as a Source Data file.

Supplementary References

1. Fogg, M. J. & Wilkinson, A. J. Higher-throughput approaches to crystallization and crystal structure determination. *Biochem Soc Trans* **36,** 771–775 (2008).
2. Liu, H. & Naismith, J. H. A simple and efficient expression and purification system using two newly constructed vectors. *Protein Expr Purif* **63,** 102–111 (2009).
3. Zimmermann, L. *et al.* A Completely Reimplemented MPI Bioinformatics Toolkit with a New HHpred Server at its Core. *J Mol Biol* **430,** 2237–2243 (2018).
4. Hildebrand, A., Remmert, M., Biegert, A. & Söding, J. Fast and accurate automatic structure prediction with HHpred. *Proteins* **77 Suppl 9,** 128–132 (2009).
5. Hynes, A. P. *et al.* Functional and evolutionary characterization of a gene transfer agent's multilocus "genome". *Mol Biol Evol* **33,** 2530–2543 (2016).
6. Dodd, I. B. & Egan, J. B. Improved detection of helix-turn-helix DNA-binding motifs in protein sequences. *Nucleic Acids Res* **18,** 5019–5026 (1990).
7. Narasimhan, G. *et al.* Mining protein sequences for motifs. *J Comput Biol* **9,** 707–720 (2002).
8. Larkin, M. A. *et al.* Clustal W and Clustal X version 2.0. *Bioinformatics* **23,** 2947–2948 (2007).
9. Sievers, F. *et al.* Fast, scalable generation of high-quality protein multiple sequence alignments using Clustal Omega. *Mol Syst Biol* **7,** 539 (2011).
10. Waterhouse, A. M., Procter, J. B., Martin, D. M. A., Clamp, M. & Barton, G. J. Jalview Version 2--a multiple sequence alignment editor and analysis workbench. *Bioinformatics* **25,** 1189–1191 (2009).
