# Supplementary material for: Identification and characterization of a direct activator of a gene transfer agent
Source: Nat Commun. 2019 Feb 5;10:595. doi: 10.1038/s41467-019-08526-1 (PMC6363796; doi:10.1038/s41467-019-08526-1)
Supplement: Supplementary file 3 — Reporting Summary [file 41467_2019_8526_MOESM3_ESM.pdf]

nature research

Corresponding author(s): Paul Fogg

Reporting Summary

Nature Research wishes to improve the reproducibility of the work that we publish. This form provides structure for consistency and transparency in reporting. For further information on Nature Research policies, see Authors & Referees and the Editorial Policy Checklist.

Statistical parameters

When statistical analyses are reported, confirm that the following items are present in the relevant location (e.g. figure legend, table legend, main text, or Methods section).

n/a	Confirmed	
☐	☒	The exact sample size (n) for each experimental group/condition, given as a discrete number and unit of measurement
☐	☒	An indication of whether measurements were taken from distinct samples or whether the same sample was measured repeatedly
☐	☒	The statistical test(s) used AND whether they are one- or two-sided<br>*Only common tests should be described solely by name; describe more complex techniques in the Methods section.*
☒	☐	A description of all covariates tested
☐	☒	A description of any assumptions or corrections, such as tests of normality and adjustment for multiple comparisons
☐	☒	A full description of the statistics including central tendency (e.g. means) or other basic estimates (e.g. regression coefficient) AND variation (e.g. standard deviation) or associated estimates of uncertainty (e.g. confidence intervals)
☐	☒	For null hypothesis testing, the test statistic (e.g. F, t, r) with confidence intervals, effect sizes, degrees of freedom and P value noted<br>*Give P values as exact values whenever suitable.*
☒	☐	For Bayesian analysis, information on the choice of priors and Markov chain Monte Carlo settings
☒	☐	For hierarchical and complex designs, identification of the appropriate level for tests and full reporting of outcomes
☒	☐	Estimates of effect sizes (e.g. Cohen's d, Pearson's r), indicating how they were calculated
☐	☒	Clearly defined error bars<br>*State explicitly what error bars represent (e.g. SD, SE, CI)*

Our web collection on statistics for biologists may be useful.

Software and code

Policy information about availability of computer code

Data collection	Agarose Gels were imaged with Quantity One software (Biorad), Fluorescence images were taken using Typhoon Scanner Control Software 1.1.0.7 (Amersham), qPCR data was collected using QuantStudio 3 Real-Time PCR System (Applied Biosystems)
Data analysis	Image analysis was carried out using ImageQuant (Amersham) and ImageJ (NCBI). Statistical analysis was done in SigmaPlot 13.0 (Systat Software). Figure graphics were created in CorelDraw 2017. Artemis and DNA plotter (Sanger Centre) were used for genome context visualization. RNAseq reads were quality checked and trimmed using FastQC version 11.0.5 and Cutadapt version 1.8.3, respectively. Kallisto version 0.43.1 was used to pseudo-align reads to the R. capsulatus SB1003 reference transcriptome, and to quantify gene expression. Differential expression analysis was performed using Sleuth version 0.29.0.

For manuscripts utilizing custom algorithms or software that are central to the research but not yet described in published literature, software must be made available to editors/reviewers upon request. We strongly encourage code deposition in a community repository (e.g. GitHub). See the Nature Research guidelines for submitting code & software for further information.

Data

Policy information about availability of data

All manuscripts must include a data availability statement. This statement should provide the following information, where applicable:

- Accession codes, unique identifiers, or web links for publicly available datasets
- A list of figures that have associated raw data
- A description of any restrictions on data availability

All data deeded to evaluate the conclusions of the paper are present in the paper and the additional data files. The complete RNAseq data was submitted to the NCBI Gene Expression Omnibus (GEO) Database. All bacterial strains or genetic constructs are securely stored locally and are available on request.

Field-specific reporting

Please select the best fit for your research. If you are not sure, read the appropriate sections before making your selection.

☒ Life sciences ☐ Behavioural & social sciences ☐ Ecological, evolutionary & environmental sciences

For a reference copy of the document with all sections, see nature.com/authors/policies/ReportingSummary-flat.pdf

Life sciences study design

All studies must disclose on these points even when the disclosure is negative.

Sample size	qPCR and GTA assays used a minimum of 3 biological replicates and 3 technical replicates, which is standard practice for this type of data.
Data exclusions	Data was generally not excluded but occasionally where a single technical replicate was substantially different than the others it was discarded as experimental error.
Replication	Technical and biological replicates were taken for each of the experiments described to allow for natural variation or experimental error. Biological replicates were also carried out at at least two different points in time.
Randomization	n/a
Blinding	Blinding was not used, and is not typically used for the small scale targeted type of experiments used here.

Reporting for specific materials, systems and methods

Materials & experimental systems

n/a	Involved in the study	
☒	☐	Unique biological materials
☒	☐	Antibodies
☒	☐	Eukaryotic cell lines
☒	☐	Palaeontology
☒	☐	Animals and other organisms
☒	☐	Human research participants

Methods

n/a	Involved in the study	
☒	☐	ChIP-seq
☒	☐	Flow cytometry
☒	☐	MRI-based neuroimaging
